# Ruminant Milk-Derived Extracellular Vesicles: A Nutritional and Therapeutic Opportunity?

**DOI:** 10.3390/nu13082505

**Published:** 2021-07-22

**Authors:** Siew Ling Ong, Cherie Blenkiron, Stephen Haines, Alejandra Acevedo-Fani, Juliana A. S. Leite, Janos Zempleni, Rachel C. Anderson, Mark J. McCann

**Affiliations:** 1 Smart Foods Innovation Centre of Excellence, Te Ohu Rangahau Kai, AgResearch Ltd., Massey University Campus, Palmerston North 4410, New Zealand; rachel.anderson@agresearch.co.nz; 2 Department of Molecular Medicine and Pathology, Faculty of Medical and Health Sciences, University of Auckland, Auckland 1051, New Zealand; c.blenkiron@auckland.ac.nz; 3 Auckland Cancer Society Research Centre, University of Auckland, Auckland 1051, New Zealand; 4 Beyond Food Innovation Centre of Excellence, AgResearch Ltd., Lincoln 7674, New Zealand; stephen.haines@agresearch.co.nz; 5 Riddet Institute, Massey University, Palmerston North 4442, New Zealand; a.acevedo-fani@massey.ac.nz (A.A.-F.); J.Leite@massey.ac.nz (J.A.S.L.); 6 Department of Nutrition and Health Sciences, University of Nebraska-Lincoln, Lincoln, NE 68583, USA; jzempleni2@unl.edu

**Keywords:** milk, extracellular vesicle, exosome, ruminant, MISEV

## Abstract

Milk has been shown to contain a specific fraction of extracellular particles that are reported to resist digestion and are purposefully packaged with lipids, proteins, and nucleic acids to exert specific biological effects. These findings suggest that these particles may have a role in the quality of infant nutrition, particularly in the early phase of life when many of the foundations of an infant’s potential for health and overall wellness are established. However, much of the current research focuses on human or cow milk only, and there is a knowledge gap in how milk from other species, which may be more commonly consumed in different regions, could also have these reported biological effects. Our review provides a summary of the studies into the extracellular particle fraction of milk from a wider range of ruminants and pseudo-ruminants, focusing on how this fraction is isolated and characterised, the stability and uptake of the fraction, and the reported biological effects of these fractions in a range of model systems. As the individual composition of milk from different species is known to differ, we propose that the extracellular particle fraction of milk from non-traditional and minority species may also have important and distinct biological properties that warrant further study.

## 1. Introduction

Milk is the only food that has evolved to meet the nutritional needs of newborns, supporting growth and development while also being a significant source of nutrients in adults [1,2,3]. The domestication of livestock was a pivotal step in the consumption of non-human milk which has become a substantial source of essential nutrients in many diets globally [4,5,6]. To meet this demand, the production of milk increased from 708 million tonnes in 2009 to 883 million tonnes in 2019, with cow and buffalo milk accounting for 81% and 15% of production, respectively (Supplementary Table S1) [7].

In early life, major milk components such as lactose (energy source), minerals (musculoskeletal development), and high-value biological proteins provide essential nutrition [8,9]. Milk consumption throughout life can also address malnutrition and can represent a significant proportion of overall nutrient intake in developing nations [10].

While the milk macro- and micronutrient composition is largely well established, there is considerable interest in milk-derived extracellular vesicles (EVs) and their cargoes as a source of nutrients in the classical sense, such as nucleosides, and amino acids, or as a nutritional component that influences biological functions by regulating biochemical pathways and/or interactions with the host’s gut microbiome [11,12,13,14,15,16,17,18]. Evolutionary theory suggests that milk-derived EVs and their cargoes must have a biological purpose to justify the metabolic cost required to produce them during lactation.

**Figure 1:**
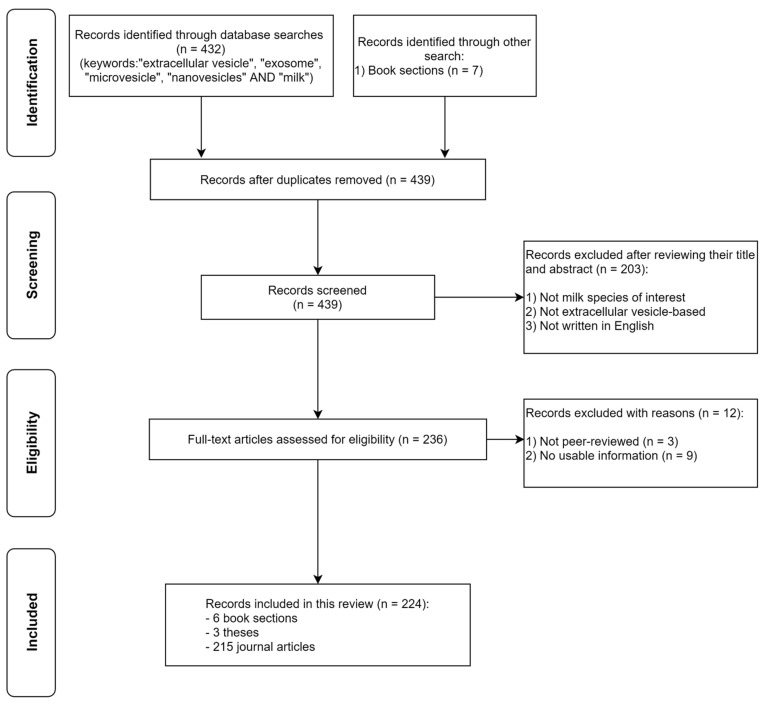
Schematic diagram for selection of included studies.

Our review covers the following: a summary of the nutritional composition of the types of milk that have been used to study milk-derived EVs, the nature and composition of these vesicles and their cargoes, the evidence for their stability and uptake in the gastrointestinal tract, their reported biological effects, and some of the key challenges in using them for studies. Methods used to identify peer-reviewed studies are shown in Figure 1. Our review excludes any studies on plant-derived milk alternatives.

## 2. Nutritional Composition of Milk

Milk from minority dairy species, i.e., not cow milk, is more widely consumed in regions with a harsh environment which requires animals with specific adaptations [9]. It has generally not been studied in as much detail for nutrition or bioactivity as cow milk despite the evidence of substantial compositional differences in the different types of proteins, lipids, micronutrients, and bioactive components between milk from different species. The macronutrient composition of milk from different mammals has been extensively studied and is readily available in the public domain. A list of the milk composition from different mammals is collated in Table 1, based on the different quantification methods used and data reporting across different databases (the conversion can be found in Supplementary Table S2).

### 2.1. Carbohydrate

Lactose is the primary carbohydrate in milk, providing 30% to 60% of energy depending on the species of milk [10,30,31,32]. It also enhances intestinal mineral absorption (e.g., calcium, sodium, magnesium, and phosphorus), utilisation of vitamin D, and stool softness [4,10,30,33]. Hydrolysis of lactose by the enzyme lactase into a simpler form of sugars is essential for intestinal absorption in humans [10,34]. Lactase deficiency contributes to the fermentation of lactose in the colon by microorganisms, producing gases (hydrogen, carbon dioxide, and methane), organic acids (acetic, butyric, and propionic acid), and excess water in stool, leading to uncomfortable bowel activity such as diarrhoea, flatulence, and bloating [1,10,35,36].

Many forms of oligosaccharides are also present in milk, contributing to the gut microbiome diversity in infants [17]. In humans, milk oligosaccharides are the third most abundant macronutrients (7 to 20 g/L) after lactose and lipids [31,37], but in other mammals, the milk oligosaccharides content is 10 to 100 times lower [37].

### 2.2. Fat

Milk fat occurs in emulsified droplets known as milk fat globules (MFGs) that are mainly triacylglycerols (97–98% of total lipids by weight, including a large number of esterified fatty acids and phospholipids), as well as proteins and fat-soluble vitamins [10,38]. The tri-layered phospholipid membrane of an MFG is designed to protect its contents from lipolysis and oxidation [38]. The roles of milk lipids and MFGs in health have recently been reviewed [39,40]. German and Dillard [41] reviewed the composition, structure, function, absorption, and bioactivity of human and cow milk lipids, noting the importance of considering the role of MFGs.

In general, the lipid composition of milk fat also differs from one species to another. Zou et al. [42] compared the lipid composition of five mammalian milks (cow, buffalo, donkey, sheep, and camel) to human milk by evaluating the degree of the chemical similarity of the samples. This showed that although the total fatty acid composition of certain non-human milks was highly similar to that of human milk (e.g., 96.4% similarity in sheep’s milk), there were substantial differences when it came to the individual chemical species (e.g., only 20.2% similarity in polyunsaturated fatty acids of sheep’s milk). Devle et al. [43] measured the fatty acid profiles in the milk of three ruminants (cow, goat, and sheep) and two non-ruminants (donkey and horse) and found a substantial diversity in the occurrence and abundance of them between species and their degree of correlation with health attributes.

### 2.3. Protein

Depending on the species, there is between 1.2 and 5.6 g of protein per 100 g of milk. Caseins (αs1-, αs2-, β-, and κ-casein) are the most abundant insoluble proteins in milk [44,45]. The soluble proteins in the whey fraction of milk mainly consist of soluble milk serum proteins (β-lactoglobulin, α-lactalbumin, immunoglobulins, serum albumins, etc.), proteose peptones (casein-derived low-molecular weight peptide and proteose peptone component 3), and membrane proteins (i.e., milk fat globule membrane (MFGM)) [10,44,46,47].

The unique ability of milk caseins to form macromolecule aggregates (casein micelles) with minerals such as calcium and phosphorus improves the bioavailability, delivery, and intestinal absorption of these minerals [4]. The industrial importance of ruminant milk proteins in cheese production and secondary transformation products has led to the extensive study of these components, such as the proteomic analysis of several forms of milk: as a whole [45], the whey fraction [48,49], and sub-fractions of whey such as caseins [50] and MFGMs [51,52,53,54].

Roncada et al. [55] reviewed advancements and challenges in the proteomic analysis of milk from farm animals, together with an overview of the different components in the milk fractions. Similarly, Malacarne et al. [56] systemically reviewed the composition of horse, human, and cow milk from the perspective of protein and lipid fractions, proposing that the nourishment provided by horse milk is more similar to human milk than that provided by cow milk.

### 2.4. Micronutrients

Micronutrients are essential nutrients that cannot be synthesised by humans and must be provided through our diet or other means [57]. The consumption of two to three servings of milk or milk products provides the required nutrient intakes for several important micronutrients (calcium, magnesium, selenium, riboflavin, vitamin B12, and pantothenic acid) [10]. Milk has comparatively fewer absorption inhibitors (e.g., oxalate and phytates) than other foods, which improves the bioavailability and absorption of these micronutrients [8,10].

The major milk minerals, calcium and phosphorus, which are required for optimal bone health are more bioavailable due to the mineralisation of casein micelles in both the insoluble organic colloid and mineral forms [58]. Medhammar et al. [9] highlighted the differences between the mineral profiles of different milk species, with moose and reindeer milk having the highest concentration of most essential minerals, and horse and donkey milk having the lowest. Milk also provides water- and fat-soluble vitamins due to the dual-phase matrix of lipid micelles suspended in the aqueous environment. Milk vitamin profiles are broadly consistent, with vitamin C having the highest concentration, and vitamins B12 and D having the lowest concentrations, with some species differences [9,59]. Graulet [57] reviewed the role of ruminant milk, with an emphasis on cow’s milk, in meeting the required vitamin consumption by humans.

### 2.5. Other Milk Components

Admyre et al. [60] identified the presence of immune-modulatory exosomes in human milk which led to research into how exosomes (one type of EV) and their cargoes may have a role in inter-cellular, inter-individual, or inter-species communication. There has been substantial interest in milk-derived EVs as a novel bioactive fraction of milk [11,12,16,61,62,63]. However, the majority of research has focused on human or cow’s milk, and not minority dairy species, and therefore this paper reviews the key technical challenges and reported biological activities of ruminant and pseudo-ruminant milk-derived EVs.

## 3. Milk-Derived EVs

According to the Minimal Information for Studies of Extracellular Vesicles 2018 (MISEV2018) guidelines, “extracellular vesicle is the generic term for any particle naturally released from the cell that is delimited by a lipid bilayer and cannot replicate, i.e., do not contain a functional nucleus” [64]. Due to historical differences in how these vesicles were isolated, characterised, and named, the guidelines recommend using the term “extracellular vesicle” instead of other terms such as “exosome” or “microvesicle”, except when the biogenesis or release pathway is investigated [64]. However, in this review, the terminology used in the original paper cited will be used. The MISEV guidelines provide experimental and reporting guidelines specific to the field of EVs [64,65,66], and several curated public knowledgebases promote the transparency and reproducibility of EV experimental studies [67,68,69,70,71,72,73,74,75]. Recent advances in the use of flow cytometry to study EVs have led to a standardised experiment and reporting framework (MIFlowCyt-EV) [64,76,77].

EVs are heterogenous populations that are categorised based on their biogenesis pathway. In brief, exosomes (~30 to 150 nm) originate from the intraluminal vesicles via the endosome trafficking pathway, while microvesicles (100 nm to 1 µm) result from direct budding from the plasma membrane of the parental cell, and apoptotic bodies (1 to 5 µm) are shed from cells undergoing apoptosis [78,79]. To date, most of the available methodologies cannot isolate a pure subpopulation of EVs; therefore, a defined mixed population is widely used for studies. The progress in understanding EV biology in the context of inter- or intra-species signal mediators, due to their diverse cargo (mRNA, miRNA, protein, etc.), has spawned a growing interest among the research community. An in-depth review of EV heterogeneity [78] and cell biology [80] proposed a need for a clear definition of the different subpopulations, based on cargo composition, trafficking pathways, and biological functions. Several other detailed reviews focused on other aspects of EVs such as biogenesis [81,82], delivery or target mechanisms [83,84,85], and current advances in knowledge [86]. The majority of publications on EVs have been focused on human growth, development, homeostasis, and disease progression, and several other reviews of milk-derived EVs are summarised in Table 2.

### 3.1. Isolation of Milk-Derived EVs

From complex biofluids to simpler in vitro cell culture media, different isolation methods may be employed to minimise the presence of unwanted artefacts which could jeopardise the downstream analysis. Review articles or book chapters on EV isolation techniques are readily available in the literature, from providing a brief overview [87,88,89,90,91,92] to a comprehensive discussion [93,94,95,96].

EVs’ isolation relies on their separation from contaminants such as proteins and other particles through the use of known biophysical and/or biochemical properties: size, buoyant density, surface charge, surface molecules’ expression and their composition. Several articles dedicated to a specific scope of isolation techniques are worth mentioning. Li et al. [97] discussed the different isolation strategies for human biofluid-derived EVs which have been employed in mass spectrometry (MS)-based proteomic studies for the past decade (2009–2019). Another review article highlighted the usefulness of size exclusion chromatography (SEC) in EV isolation, given that this approach is highly scalable and adaptable while maintaining the EVs’ characteristics [98]. A three-step filtration protocol comprising dead-end filtration, tangential-flow filtration, and track-etched membrane filtration was proposed by Heinemann and Vykoukal [99] to provide an approach to concentrate and fractionate samples with minimal forces applied on EVs. The progression in microfluidics-based platforms in the past decade has enabled the rapid separation of EVs from small sample volumes. A review by Meng et al. [100] highlighted the interesting advancements in the microfluidic separation of EVs based on the different separation principles.

Characterisation of isolated EVs still largely uses immunochemical (e.g., ELISA, Western blot), MS-based, and optical (e.g., nanoparticle tracking analysis (NTA), microscopy, and flow cytometry) methods. However, any of the single aforesaid detection approaches may not be sufficient to address the issues of specificity, efficiency, and consistency in EV detection. More often, multiple detection approaches are employed within the research community when it comes to EV characterisation. The progression in analytical sciences has pushed for the development of new and innovative instruments to meet the abovementioned challenges. Recent review articles have summarised the emerging new technologies available that are specifically developed for EV characterisation [94,101,102,103,104].

Methods for the isolation and characterisation of milk-derived EVs have no significant differences compared to those for isolation from other biofluids or cell culture media; thus, any protocol, technique, or technology for isolation of EVs of different origin can also be used for those from milk. The only difference between these types of samples is the unwanted artefacts present in different biofluids (e.g., lipoproteins in blood serum, or casein aggregates in milk). A simple method for isolating EVs from breast milk was described by Wang [50], which only requires a proprietary precipitation reagent (ExoQuick), a benchtop centrifuge, and a few common lab consumables; however, this method isolates a crude preparation of EVs. Several other approaches have been developed and used to study EVs (Table 3).

### 3.2. Protein Composition of Milk-Derived EVs

The application of MS-based proteomic profiling and protein quantification has been of substantial significance in EV research to allow the identification and quantification of EV proteomes from various cultured systems, organs, body fluids, or plants. Several review articles provide a high-level overview of the MS-based methodological approaches widely used in EV studies [79,120,121,122,123]. MS-based proteomic quantitative analysis can be achieved with either a labelled (e.g., isobaric tags for relative and absolute quantification (iTRAQ); stable-isotope labelling of amino acids (SILAC)) or label-free approach which quantifies proteins based on their spectral intensity or counts [79,120]. Data generated from MS consist of large datasets with functional analysis of these data needed for the identification of biological processes, which includes the Gene Ontology (GO) term annotation, enrichment analysis, and/or pathway analysis [124].

The early discovery of several EV-enriched protein markers (tetraspanins, heat shock proteins, annexins, etc.) from isolated EVs derived from in vitro cell models using MS-based proteomic characterisation occurred in the early 2000s [125,126,127]. Admyre et al. [60] first reported the investigation of the mammalian milk-derived EV proteome using a tandem MS approach to verify several important EV protein markers (tetraspanins, heat shock proteins, MUC-1, etc.) from the human colostrum and mature breast milk-derived exosomes, respectively. Building on this, Reinhardt et al. [128] identified 2107 proteins in a comprehensive study of cow milk-derived exosomes by utilising two-dimensional liquid chromatography-based separation coupled with tandem mass spectrometry. These studies led to several characterisation papers, summarised in Table 4 [128,129,130,131,132,133,134,135,136,137]. The literature demonstrates that milk-derived EVs have a distinctive proteome compared to other milk fractions and that a significant proportion of these proteins have reported immune-regulatory properties.

### 3.3. Lipid Composition of Milk-Derived EVs

As different EV subtypes (exosomes, microvesicles, and apoptotic bodies) are categorised, in part, based on their respective biogenesis pathways, the membrane lipid composition of EVs resembles that of the parent pathway [93,145]. Understanding the lipid composition of EVs, such as sphingolipids, ceramides, phosphatidylserine, and the lipid raft component cholesterol, is an essential part of their biology, biogenesis, and biological function [121,146]. Several of the analytical chromatography and mass spectrometry techniques routinely used in EV proteomics have also been used for the qualitative and quantitative assessment of EV lipids. The challenges, limitations, and current knowledge of EV lipidomics have been reviewed elsewhere [121,145,147,148,149,150].

There are particular challenges in isolating lipids from milk-derived EVs due to the co-isolation of milk lipids (i.e., MFGs), and in milk EV isolates with a high triacylglycerol content (TAG), since MFGs contain a greater amount of TAGs in their core than EVs [38,119], potentially interfering with accurate EV lipidomic studies. In this review, MFG lipidomic studies are not included because the biophysical properties (tri-layered membrane) and cargoes of MFGs are distinctly different from those of EVs.

To date, two studies have specifically examined the lipid composition of EVs [119,151]. Blans et al. [119] successfully applied size exclusion chromatography to human and cow milk samples to isolate distinct fractions of EVs and MFGs; these were partly characterised by the notably higher TAG-to-cholesterol ratio in human and cow MFGMs in MFGs when compared to EVs. The authors also reported a higher proportion of sphingomyelin, phosphatidylserine (PS), and phosphatidylcholine (PC), and a lower proportion of phosphatidylethanolamine (PE) in EVs compared to MFGs. Yassin et al. [151] reported concentrations of ~10 to 15 µg/mL of phosphatidylinositol, PS, and PE, and ~20 to 25 µg/mL of PC in dromedary milk exosomes which were consistent during different lactation periods.

Phospholipids, such as those reported in EVs, have been associated with beneficial health effects [152,153,154,155,156]. We recognise that there is a knowledge gap in the understanding of the lipid composition of mammalian milk-derived EVs, which is essential to understanding the biology of their function and biogenesis mechanisms [157].

### 3.4. Nucleic Acid Composition of Milk-Derived EVs

Milk-derived EVs contain nucleic acid cargoes, proposed to be derived from mammary epithelial cells, encased within the cytosol of a lipid bilayer vesicle [158,159]. Studies characterising the milk-derived EV transcriptome are summarised in Table 5.

Of interest is the presence of microRNA (miRNA) in milk; these are short nucleic acids of ~22 nucleotides and are known for their role in post-transcriptional regulation. In milk, these miRNAs are present in two main forms: bound to RNA-binding proteins, or encapsulated in EVs [12]. The abundance of miRNAs in milk has generated substantial interest and research into whether these miRNAs are bioavailable and bioactive. Many studies have focused on the potential involvement of milk-derived EV miRNA in inter-cellular crosstalk, inter-individual communication (breastfeeding), and cross-species communication (due to human consumption of other mammalian milk throughout adulthood). However, it is noted that the concept of ingested miRNA from another species surviving digestion and being absorbed in sufficient quantities to elicit a quantifiable biological effect remains to be convincingly shown, despite several promising studies [109,160,161,162].

Several review articles on milk-derived EV nucleic acid cargoes are available, which include their functional implications [61,163,164] and future applications [165,166]. Additionally, with development in the application of next-generation sequencing (NGS), several nucleic acid profiling studies (with an emphasis on miRNA) have reported their findings from milk-derived EVs in humans [167,168,169,170,171,172,173], cows [158,174,175,176,177,178,179], pigs [129,170,180,181], sheep [182], and buffaloes [183]. In silico insights from these data suggest potential regulation of several key pathways, but for the most part, these predictions have not been validated in vitro or in vivo.

However, the presence of miRNA in milk-derived EVs suggests that they have a potential role as natural or modifiable therapeutic agents to improve or enhance human and animal health. For instance, there are studies evaluating the milk-derived EV transcriptome for use as nanotherapeutic agents [184,185,186], as disease biomarkers [158,179,187], differential mediators [188,189], and as a health assessment tool for lactating animals [175]. Conversely, the role of milk-derived EVs as a functional regulator has also generated concerns because continuous consumption of dairy may contribute to the pathogenesis of common Western diseases such as type 2 diabetes mellitus, allergies, and cancers [13,190,191,192,193,194].

In brief, there are two broad schools of thought regarding the specific role of milk-derived miRNAs in postnatal development: (1) the functional hypothesis, which proposes that these miRNAs are purposefully transferred by the parent to the offspring to exert meaningful epigenetic regulatory functions in the infant’s development, and (2) the nutritional hypothesis, which proposes that the degradation of miRNAs in the gut during digestion into nucleotides means that they are only nutritional “building blocks” for the infant only and do not exert any meaningful regulatory functions [191].

The studies listed in Table 5 show that: (1) RNA (especially miRNA) is present in milk-derived EVs and other extracellular particles, (2) some of these miRNAs are conserved between species, (3) some of these miRNAs are specifically found in extracellular particles, and (4) biological dysfunction, such as disease, can alter miRNA abundance. On the assumption that conservation of the miRNA sequence implies conservation of function, much of the research into the biological effects of milk-derived EVs has focused on their miRNA cargoes and their effects on immune regulation. These studies, and others, are reviewed later.

**Table 5:** Major findings of nucleic acid studies conducted on EVs of different mammalian milk used to characterise the RNA composition of milk-derived EVs and exosomes.

Species	Technique	Findings	Ref.
		**Extracellular Vesicle**	
Human	NGS	Total of 1523 miRNAs identified with more than one read in 70% of samples from the Faroe Islands cohort (364 mothers).	[195]
Human	qPCR	Total of 55 lncRNAs identified with 11 lncRNA detected in >50% of the breast milk samples and 5 in >90%. The authors suggested the packing of highly correlated lncRNAs is regulated by the same pathway.	[196]
Human	NGS	Total of 5 miRNA stably expressed in all groups. Total of 4 (probiotic^+^) and 5 (atopic dermatitis^+^) miRNAs differentially expressed. No evidence of maternal probiotic ingestion altering miRNA abundance, unlikely for probiotic protective effect to be transferred to the infants.	[172]
Human + Pig	qPCR, NGS	Identified 309 (human) and 218 (pig) mature miRNAs. In silico analyses demonstrated evolutionary conservation of the top 20 most abundant miRNAs between human, cow, pig, and panda.	[170]
Cow	qPCR, NGS	Identified more than 200 cow milk-derived EV miRNAs.	[109]
Cow	qPCR, NGS	Enrichment of small RNA profiles in 4 fractions (12 k, 35 k, 70 k, and 100k× *g*). Distinct differences in small RNA biotypes between fractions.	[174]
Cow	qPCR, NGS	Total circRNAs: 39,276 identified, with 17,169 unique to *Staphylococcus aureus*-infected cows. Demonstrated the selective circRNA packaging mechanism regulated by the infection.	[197]
Cow	Microarray	mRNA profiles are altered by viral load and lactate dehydrogenase concentration.	[187]
Cow	qPCR, NGS	Total of 276 miRNAs identified with 9 differentially expressed between forage-fed and non-forage fibre source-fed cows.	[175]
Cow	qPCR	Demonstrated an enriched subset of miRNAs in EVs prepared at 12,000 and 35,000× *g*, which were traditionally discarded during preparation.	[133]
Cow + Sheep	qPCR, NGS	Identified 685 miRNAs (601 novel) in sheep samples. In silico comparison of the top 20 expressed miRNA in both milks that have immune-related functions.	[182]
		**Exosome**	
Human	qPCR, NGS	Identified 221 and 48 mature miRNAs (fresh and 4-week-old milk stored at 4 °C, respectively) detected in 1 mL samples. No reliable detection of miRNAs in infant formula.	[167]
Human	qPCR, NGS	Total miRNAs: 631 detected, with 208 novel miRNAs. Total of 9 miRNAs differentially abundant in type 1 diabetes samples.	[168]
Human	qPCR, NGS	Identified 602 miRNAs with 59 miRNAs that are immune-related. Demonstrated resistance and stability of exosomal miRNAs against harsh conditions.	[173]
Human and Pig	*In silico*	Reported the presence of plant miRNA in both human and pig milk exosomes based on publicly available sequencing data.	[198]
Cow	Qpcr	Demonstrated the bioavailability of cow milk exosomal miRNAs in human plasma without eliciting a cytokine response ex vivo (human PBMCs).	[199]
Cow	PCR, NGS	Total miRNAs: 290 detected, with 69 novel miRNAs. Total of 37 miRNAs differentially expressed due to infection. The predicted target genes for 2 miRNAs highly expressed in infected samples, bta-miR-378 and bta-miR-185, were functionally validated with target genes.	[200]
Cow	qPCR, NGS	Total miRNAs: 1472 detected, with 480 novel miRNAs. Total of 18 miRNAs differentially expressed due to mastitis. Presented miRNA expression profiles of both healthy and infected cows. bta-miR-223 and bta-miR-142-5 were considered potential early mastitis detection targets.	[158]
Cow	qPCR	Reported the effects of fermentation on the expression of miR-29b (unaffected) and miR-21 (significantly reduced by fermentation).	[201]
Cow	qPCR, Microarray	Microarray profiling of miRNA (79) and mRNA (19,320) on exosome obtained via ultracentrifugation and its supernatant.	[178]
Cow	NGS	Total miRNAs: 417 detected, with 303 novel miRNAs. Two differential expression analyses revealed 6 miRNAs with significant differential presence. Total of 2 miRNAs were proposed as potential biomarkers for early infection.	[179]
Cow and Buffalo	NGS, in silico	Total miRNAs: 558 detected in all species (buffalo, cow, pig, human, and panda), with the top 10 highly expressed miRNAs conserved across species. Total of 48 miRNAs were differentially expressed in buffalo, compared to other species.	[183]
Pig	qPCR, NGS	Total mRNAs: 16,304 detected, with 2409 novel mRNAs. A random selection of 14 mRNAs among the top 50 was further confirmed using qPCR.	[129]
Pig	qPCR, NGS	Total miRNAs: 491 detected, with 315 novel miRNAs.	[180]
Pig	qPCR, NGS	Total pre-miRNAs: 180 detected, with 40 novel pre-miRNAs, corresponding to 237 mature and 234 unique miRNAs. Immune-related miRNAs are most abundant in colostrum.	[181]
Camel	qPCR	Stable expression of the casein family genes between mid and late lactation periods.	[151]
		**Microvesicle/Nanovesicle**	
Human	qPCR Microarray	Total of 281 miRNAs detected. Expression of miR-181a and miR-17 was detected in CD63-positive human milk exosomes.	[202]
Cow	qPCR	Six different cow colostrum exosome isolation methods were compared. Method 2 (conventional: differential centrifugation) had the highest purity and greatest amount of microvesicular miRNAs quantified.	[203]
Cow	qPCR	Identification of selected mRNA and miRNA in microvesicles, unaffected by acidification, and in vitro transfer of RNA from samples.	[204]
Buffalo	qPCR	The expression of 6 nanovesicular miRNAs from three biofluids was evaluated, and 2 of them (miR-21 and miR-500) were reported to be stably expressed during different household storage conditions.	[205]

The terminology used is based on the reference cited, and this division reflects older thinking and is a “pool” of EVs that are responsible for the effects (circRNA, circular RNA; lncRNA, long non-coding RNA; miRNA, micro-RNA; NGS, next-generation sequencing; qPCR, quantitative PCR).

## 4. Stability and Uptake of Milk-Derived EVs

The studies reported in the previous sections described how EVs contain a range of lipids, proteins, and nucleic acids. The stability, i.e., resistance to degradation due to processing or digestion, of milk EVs and cargoes, and how they are taken up by the recipient cells have been studied in cows [159,160,169,177,178,201,204,206,207,208,209,210,211,212,213,214,215], humans [167,171,173,202,216,217], goats [218], and buffaloes [205]. These studies are summarised in Table 6.

These studies show that the structure of EVs protects them against harsh conditions, such as low pH, temperature variations, or high concentrations of RNase. This capacity to resist degradation and digestion underpins the study of their potential biological effects, either as nutrient delivery or as drug delivery vehicles.

## 5. Biological Effects of Milk-Derived EVs

The previous sections indicate that milk-derived EVs may contain bioactive components, which are protected against degradation and digestion. The studies to date that have focused on the biological effects of milk-derived EVs are highlighted in Table 7. The majority of these studies used human or cow milk EVs, and there is a clear knowledge gap regarding whether milk from other species has similar or different effects.

The studies researched the effects of EVs and exosomes on the gut microbiota [201,223,225], on the use of a delivery vehicle [111,159,184,208,213,226,227,228,229,230,231], in the immune response [60,178,185,203,217,232,233,234,235,236,237], in diseases such as cancer, [188,189,209,228,231,236,238,239,240,241,242,243,244,245,246,247], and in other aspects of cell biology [129,162,248,249,250,251,252,253]. These studies show that milk-derived EVs can have meaningful biological effects in the model systems used, forming a basis for future research.

Whether these effects in animal and in vitro models translate into humans is unclear. A question remains concerning whether the effects are solely due to EVs and their cargoes or also due to other variable contaminants (e.g., RNA-binding proteins) in the vesicle preparations used in the published studies. The increased rigour and reporting required to comply with the MISEV guidelines are intended to enable more thorough validation of EV research.

**Table 7:** Major findings of studies on the biological effects of mammalian milk-derived EVs.

Species	Findings	Ref.
	**Extracellular vesicle**	
Human	Protective effect in vitro (MA-104 and Hep-2 cell lines) against human rotavirus and respiratory syncytial virus.	[254]
Human	In vitro (HFF-1 cell line) antiviral activity against human cytomegalovirus via inhibition of viral replication.	[138]
Human	Antiviral activity against Zika and Usutu in vitro (Vero cell line).	[255]
Human	Coagulant potential of human milk, owing to the presence of tissue factor (TF)-exposing EVs, but not found in cow milk.	[256]
Human	Protective effect against experimental-induced NEC in vitro (IEC-6 and FHs 74 Int cell lines) and in vivo (Sprague Dawley pups).	[257]
Human + Cow	Attenuation of inflammatory cytokine expression and nuclear factor (NF)-κB activation in vitro (LPS-stimulated RAW 264.7).	[258]
Cow	Promotion of osteogenesis via proliferation and differentiation of osteoblasts in vitro (Saos-2 cell line) and in vivo (Sprague Dawley rats).	[259]
Cow	Improved small intestinal dysfunction in malnutrition C57BL/6J mouse model.	[260]
Cow	Enhancement of curcumin cell uptake and permeability in an intestinal model in vitro (Caco-2 cell line).	[261]
Cow	Osteoprotective effects in vivo (BALB/c and C57BL/6 mice), and decreased the RANKL/OPG ratio in vitro (MLO-Y4 cell line).	[249]
Cow	Induction of phenotypical changes in hPAEC and NRCM cell lines.	[109]
Cow	Modulation of gut microbiota composition, SCFA profiles, and enhancement of intestinal immune regulation by EVs in vitro (RAW 264.7 cell line) and in vivo (C57BL/6J mice).	[225]
Cow	Differential improvements in DSS-induced colitis of two EV subsets via different mechanisms in vivo (C57BL/6J mice).	[188]
Cow	Modulation of agricultural dust-induced lung inflammation by EVs in vitro (MH-S cell line) and in vivo (C57BL/6J mice).	[232]
Cow	Demonstrated sonication effects on EV skeletal muscle biomarkers in vivo (Fischer 344 rats).	[262]
Cow	Biocompatibility and potential use as a non-immunogenic delivery vehicle of EVs in vitro (RAW 264.7) and in vivo (ICR mice).	[111]
Cow	Demonstrated EVs do not cause genotoxicity and contain bioactive TGF-β in vitro (NIH/3T3 cell line), and EVs facilitate differentiation of naive T cells into pathogenic Th17 cells (ex vivo DBA/1J mice). The panel of toxicology studies found differences in toxicological profiles in vitro (HL-60, RAW 264.7, and CHO-K1 cell lines) and ex vivo (human blood).	[234]
Cow	Increased osteocytes number and osteoblast differentiation in vivo (DBA/1J mice), and increased osteoblast differentiation transitioning into osteocytes in vitro (human MSCs).	[263]
Cow	EVs significantly delayed arthritis development in vivo (IL-1Ra^-/-^ and DBA/1J mice). EV uptake demonstrated via ex vivo (mouse ileal cells and splenocytes) and in vitro (RAW 264.7 cells).	[238]
Cow	EVs contain bioactive TGF-β in vitro (NIH/3T3 cell line), and EVs facilitate differentiation of naive T cells into pathogenic Th17 cells (ex vivo DBA/1J mice).	[233]
	**Exosome**	
Human	Protective effect of both raw and pasteurised exosomes against NEC in vivo (C57BL/6 mice) and ex vivo (neonatal mice intestinal organoids).	[239]
Human	Demonstrated that miR-148a influenced the proliferation, morphology, and protein expression of transformed cells more so than normal cells in vitro (LS123 and CCD841 cell lines). The role of miR-148a was validated using a knockdown model in vitro (293T cell line).	[189]
Human	Protection against H_2_O_2_-induced oxidative stress in NEC in vitro (IEC-6 cell line).	[240]
Human	Showed uptake of exosomes, increased expression of miR-148a, and decreased expression of DNA-methyltransferase 1 in vitro (CRL-1831, K-562, and LIM1215 cell lines).	[250]
Human	TGF-β2 influences epithelial–mesenchymal transition in vitro (MCF-7 and MCF 10A cell lines).	[244]
Human	Inhibition of HIV-1 viral transfer to CD4+ T cells ex vivo (human MDC organoids).	[235]
Human	The abundance and composition of exosomes vary due to lactation stage, maternal sensitisation, and lifestyle, which influence the regulation of the allergic outcome in the child.	[247]
Human	The presence of MHC classes I and II, CD63, CD81, and CD86 on exosomes, inhibition of anti-CD3-induced cytokine production, and an increase in Foxp3^+^ CD4^+^ CD25^+^ T regulatory cells ex vivo (human PBMCs).	[60]
Cow	The loading of miRNA (hsa-miR-148a-3p) as a nanocarrier in vitro (HepG2 and Caco-2 cell lines).	[264]
Cow	Activation of immune cells ex vivo (human PBMCs) under inflammatory conditions.	[265]
Cow	Restoration of small intestinal epithelial architecture and barrier function in malnourished C57BL/6J mice.	[266]
Cow	Exosomes influence macrophage proliferation and protect against cisplatin-induced cytotoxicity in vitro (RAW 264.7 cell line).	[236]
Cow	Exosomes have cytoprotective and anti-inflammatory activity in ulcerative colitis in vivo (Kindlin 2^−/−^ mice).	[237]
Cow	Protective effects in vitro (IEC-6 cell line) against oxidative stress.	[267]
Cow	Osteoporosis prevention in in vitro (MC3T3-E1 and RAW 264.7 cell lines) and in vivo (C57BL/6J mice) models. Additionally, the restoration of gut microbiota affected by osteoarthritis.	[268]
Cow	Exosomes can be used as an siRNA delivery vehicle in vitro (A549 cell line) and have anti-tumour activity against lung tumour xenografts in vivo (athymic nude mice) and in vitro (MDA-MB-231, MCF7, A549, H1299, PANC-1, Mia PaCa-2, and A2780 cell lines).	[227]
Cow	The use of exosomes as an oral delivery vehicle in xenografts, which enhanced gut absorption and retention involving neonatal Fc receptor in vivo (Balb/c mice, CT26 cells).	[229]
Cow	Enhanced goblet cell activity, improved response against NEC in vivo (C57BL/6 mice), and increased mucin production in vitro (LS174T cell line).	[242]
Cow	Bilberry anthocyanins encapsulated in exosomes were preferentially taken up by colonic cancer cells in vitro (HCT 116, HT-29, CCD-18Co cell lines), and therapeutic enhancement with encapsulated anthocyanins showed no significant differences in vivo (C57BL/6J mice).	[228]
Cow	Depletion in dietary milk exosomes and their miRNA aggravates irritable bowel disease in vivo (*Mdr1a*^−/−^ mice).	[269]
Cow	Exosomes have a minimal effect on skeletal muscle biology in vivo (C57BL/5 mice), suggesting that other tissues may be the targets of exosomes.	[245]
Cow	The use of paclitaxel encapsulated in exosomes as a drug delivery vehicle in vivo (athymic nude and C57BL/6 mice).	[213]
Cow	Enhancement of skeletal muscle protein synthesis and anabolism in skeletal muscle cells independent of amino acids in vitro (C_2_C_12_ myoblast).	[246]
Cow	Resistance of exosomes to in vitro digestion and subsequent internalisation and trans-epithelial transport in vitro (Caco-2 cell line).	[159]
Cow	The effects on exosomes of in vitro fermentation using three combinations of probiotic bacteria, uptake of these exosomes, and increased proliferation due to the upregulation of ERK1/2 and p38 in vitro (IEC-6 cell line).	[201]
Cow	The use of encapsulated celastrol as a drug delivery vehicle, and anti-tumour activity against lung tumour xenografts in vivo (athymic nude mice, A549 and H1299 cell lines).	[231]
Cow	The use of both encapsulated hydrophilic and lipophilic small molecules as a delivery vehicle, with tumour targetability, cross-species tolerance, and enhanced drug efficacy compared to free drugs in vivo (athymic nude mice) and in vitro (A549, H1299, MDA-MB-231, T47D, and Beas-2B cell lines).	[212]
Cow	The uptake, transport kinetics, and presence of exosomal surface glycoproteins and inhibitors of endocytosis in vitro (Caco-2 and IEC-6 cell lines).	[209]
Cow + ASC + Coconut	Promotion of bacterial growth and alteration of gene expression in vitro (*Escherichia coli* K-12 MG1655 and *Lactobacillus plantarum* WCFS1 cultures).	[251]
Cow + Mice+ Pig	Inter-species and intra-species bioavailability and distribution of exosomes in vivo (Balb/c mice).	[223]
Cow + Yak	Higher growth efficiency in vitro (IEC-6 cell line) under hypoxic conditions when supplemented with yak exosomes rather than cow milk-derived exosomes.	[270]
Buffalo	Increased stability, solubility, and bioavailability of digested and undigested EV-encapsulated curcumin in vitro (Caco-2 cell line).	[226]
Camel	Anticancer effects, via induction of apoptosis, inhibition of oxidative stress, reduced angiogenesis, and metastasis, in vivo (albino rats) and in vitro (MCF7 cell line).	[252]
Rat	Rat milk-derived exosomes promote intestinal epithelial cell viability, enhance proliferation, and stimulate intestinal stem cell activity in vitro (IEC-18 cell line).	[243]
Pig	Protective effect against deoxynivalenol (DON)-induced intestinal damage in vivo (Kunming mice) and in vitro (IPEC-J2 cell line).	[271]
Pig	Protective effects of exosomes against LPS-induced effects in vivo (Kunming mice) and in vitro (IPEC-J2 cell line).	[185]
Pig	Promotion of digestive tract development, alteration in the expression of proliferation-related genes in vivo (Kunming mice), and altered cell proliferation, proliferation-related gene expression, and miRNA concentration in vitro (IPEC-J2 cell line).	[272]
Pig	Expression of miRNA during different lactation stages, and a higher uptake of colostrum-derived immune-related miRNA in vivo (piglets).	[181]
Pig + Cow	Both cow and pig milk exosomes alter serum miRNAs in vivo (piglets), and exosomal miRNA is taken up in vitro (IPEC-J2 cell line).	[162]
	**Microvesicle/Nanovesicle**	
Cow	Suitability of nanovesicles and encapsulated siRNA as a therapeutic delivery vehicle in vivo (zebrafish) and ex vivo (C57BL/6 splenocytes).	[184]
Cow	Demonstrated successful uptake of PKH67-labelled microvesicles in vitro (RAW 264.7 cell line).	[203]

The terminology used is based on the reference cited, and this division reflects older thinking and is a “pool” of EVs that are responsible for the effects (ASC, adipose-derived stem cell; DSS, dextran sulphate sodium; hPAEC: human pulmonary artery endothelial cell; MDC, monocyte-derived dendritic cell; MSC, mesenchymal stem cell; NEC, necrotising enterocolitis; NRCM, neonatal rate cardiac myocyte; PBMC, peripheral blood mononuclear cell; SCFA, short-chain fatty acid; LPS, lipopolysaccharide).

## 6. Conclusions

Not all types of milk provide the same nutritional value for inter-species consumption. Species-dependent differences are evident in the macromolecule composition (fat, sugars, etc.), vitamin and mineral content, and how it is digested after consumption [30,273,274]. Furthermore, milk has differences in its molecular composition and conservation of function that influence its specific biological value depending on the species of origin. It is reasonable to propose that EVs in milk from different species may have a differing composition that may affect their nutritional value from an EV-mediated view. Infant formula derived from cow’s milk is still the largest source of non-human infant foods worldwide, but there are areas of the world where cow’s milk is not traditionally consumed.

What nutritional effects that EVs from non-traditional and minority milk may have is poorly understood and represents a substantial gap in our knowledge. We have provided a brief summary of nutritional aspects of mammalian milk and summarised the research on milk-derived EVs of human and common mammalian livestock. We have also discussed research around therapeutic attributes, cargoes of milk-derived EVs, and techniques for working with them.

However, isolating, characterising, and assigning biological effects to milk-derived EVs are challenging due to the highly complex nature of milk as a biofluid. Careful consideration and reporting of standardised methods, i.e., MISEV guidelines, are critical to studies seeking to identify true and meaningful biological effects. The stability and bioavailability of nutrients, combined with their subtle effects (compared to pharmaceuticals), mean that any research on milk EVs needs to be carefully designed to correctly assign their functions in supporting human health.

## Figures and Tables

**Table 1:** Gross composition of milk of different mammals obtained from available food composition databases. Data are presented as an average (±standard deviation) per 100 g.

Species	Energy (kJ)	Carbohydrate (g)	Fat (g)	Protein (g)	Water (g)	Ref.
Buffalo	473 (66)	4.9 (0.4)	8.3 (1.4)	4.7 (1.3)	80.9 (2.2)	[19,20,21]
Camel	273 (42)	4.2 (1.6)	3.8 (0.4)	3.6 (1.3)	87.6 (2.2)	[19,21,22]
Cow (≥3% fat)	277 (22)	4.7 (0.5)	3.8 (0.5)	3.3 (0.2)	87.3 (0.7)	[19,20,22,23,24,25,26,27]
Cow (1–2.9% fat)	196 (14)	4.9 (0.1)	1.5 (0.4)	3.4 (0.2)	89.6 (0.3)	[19,22,23,24,25,26,27]
Cow (<1% fat)	151 (7)	4.9 (0.2)	0.2 (0.2)	3.6 (0.2)	90.3 (0.6)	[19,22,23,25,26,27]
Donkey	175	6.1	1.0	2.0	90.4	[19]
Goat (≥3% fat)	288 (39)	4.6 (0.3)	4.0 (0.7)	3.4 (0.4)	87.5 (1.5)	[20,22,23,24,25,26]
Goat (<3% fat)	212	3.9	2.4	2.7	90.2	[24]
Horse	177	5.4	1.1	2.1	91.0	[28]
Sheep	406 (16)	4.9 (0.2)	6.2 (0.5)	5.6 (0.3)	82.7 (0.6)	[22,23,26]
Human						
*Colostrum*	242 (7)	6.8 (0.3)	2.6 (0.0)	2.0 (0.1)	88.2 (0.0)	[22,23,29]
*Transitional*	267 (18)	6.7 (0.2)	3.4 (0.5)	1.5 (0.1)	87.1 (0.6)	[19,23,26]
*Mature*	293 (9)	7.3 (0.7)	4.2 (0.2)	1.2 (0.1)	87.3 (0.3)	[19,22,23,25,27]

**Table 2:** Recent review articles of milk-derived EVs.

Authors	Scope of Review	Ref.
Galley et al.	Update on the therapeutic potential of human milk-derived EVs in disease, with an emphasis on necrotising enterocolitis.	[11]
Sanwlani et al.	Discussed the mediator role of milk-derived EV crosstalk from inter-cellular to cross-species and highlighted the emerging therapeutic potential of milk-derived EVs.	[12]
Melnik et al.	Reviewed epidemiological and translational evidence on how dairy milk-derived exosomes (along with their cargo) contribute to the pathogenesis of common Western diseases.	[13]
Munir et al.	Highlighted the role of food-derived exosomes on human physiological and pathological events, as well as their potential as a therapeutic agent.	[14]
Zempleni et al.	Discussed the bioavailability and the distribution of milk-derived exosomes and their cargo (emphasis on miRNA).	[63]
de la Torre et al.	Summarised the general biophysical features and roles in health and disease of EVs. The authors also focused on human breast milk-derived exosomes in maternal and infant health, based on an in-depth discussion on two proteomic datasets of human breast milk exosome studies.	[16]
Le Doare et al.	Discussed the role of human milk microbiota, milk oligosaccharides, and EVs in the development of the infant gut microbiome and immune system.	[17]
Foster et al.	Summarised the knowledge about EVs derived from human biofluids, with emphasis on the human reproductive system.	[18]

**Table 3:** Methods used to study EVs and exosomes from milk.

Species	Methodology	Findings	Ref.
		**Extracellular Vesicle**	
Human	DC + PR	EV isolation from human milk via precipitation using ExoQuick.	[105,106]
Human	DC + top-down DG-UC, DC + bottom-up DG-UC	DC + top-down DG-UC was efficient and reproducible with a heterogeneous population of EVs (sizes and types).	[107]
Cow	UC, SEC, PR, membrane affinity column, PS-affinity isolation	SEC-based qEV column (Izon Science) yielded high purity (high EV count per mg protein) and a large amount of RNA with minimal operation time.	[108]
Cow	DC + UC, DC + EDTA + UC, DC + DG-UC	DC + DG-UC yielded the highest abundance of miRNA with EV surface protein markers.	[109]
Cow	UC, HCl or AA	EV concentration was significantly higher for samples treated with acidification, suggesting efficient removal of casein. However, acidification was reported to partially degrade EV surface proteins (i.e., CD9 and CD81). TEM images revealed a rough surface of EVs isolated with acids.	[110]
Cow	AA+UC, C + UC	AA+UC method yielded lower protein content, but EV protein markers (CD81, Rab5B, TSG101, and Hsc70) were reported to be present in high abundance. Proteome analysis revealed C/UC EV fraction contains whey proteins such as casein, albumin, lactoferrin, and lactoglobulin.	[111]
Cow	Total particles and Annexin V^+^ particles measured using flow cytometry (Canto II and Cytoflex) and NTA (NanoSight)	Significant correlation of total particle counts using Cytoflex and NanoSight and for Annexin V+ particles using Canto II and Cytoflex.	[112]
Cow + HCT 116 cell line + *Ascaris suum*	AFM-based force spectroscopy (FS)	Demonstrated an AFM-based characterisation strategy with the ability to discriminate EVs from contaminants.	[113]
		**Exosome**	
Human	Novel solid-phase extraction in tip-based format	Demonstrated successful recovery of spiked lyophilised human urine exosomes from 3 different matrices (mock urine, reconstituted non-fat milk, and foetal bovine serum).	[114]
Cow	UC, IP	IP had a better efficiency in removing casein and reduced operator time. TEM revealed precipitated exosomes had rough surfaces. Other features of exosomes isolated were not significantly different.	[115]
Cow	DC + DG-UC, DC + SEC	Increased yield and better purity of intact exosomes with DC + SEC method.	[116]
Cow	PR, UC + PR, UC + DG-UC, Filtration + UC	PR alone and Filtration + UC unsuitable due to the species difference. UC + PR was useful for rapid isolation with increased recovery. UC + DG-UC suitable for efficient purification with native form intact.	[117]
Human + Cow	DC + SEC	Evaluation of Vaswani et al. [116] on human milk. The enrichment profile of exosomes was similar to that obtained in cow milk in their previous study, suggesting the method was suitable for use on human milk.	[118]
Human + Cow	UC (milk serum) + SEC, C+ UC (fluff layer) + SEC	Isolation and characterisation of EVs from both milks compared to conventional UC.	[119]

The terminology used is based on the reference cited, and this division reflects older thinking and is a “pool” of EVs that are responsible for the effects (AA, acetic acid; AFM, atomic force microscopy; C, centrifugation; DC, differential centrifugation; DG-UC, density gradient ultracentrifugation; EDTA, ethylenediaminetetraacetic acid; IP, isoelectric precipitation; NTA, nanoparticle tracking analysis; PR, precipitation reagent; PS, phosphatidylserine; SEC, size-exclusion chromatography; UC, ultracentrifugation).

**Table 4:** Major findings of analytical techniques used to characterise the protein composition of milk-derived EVs and exosomes.

Species	Technique	Findings	Ref.
		**Extracellular Vesicle**	
Human	μLC-MS/MS	Identified 258 EV membrane surface proteins (surfaceome) that contributed to antiviral activity.	[138]
Human	nLC-MS/MS, LC-MS/MS	Identified 1963 proteins (198 novel). Construction of human milk proteome (n = 39 individual studies) found 2698 unique proteins (633 previously reported in EVs).	[130]
Human	nLC-MS/MS	Identified 73 proteins and the presence of several exosomal protein markers.	[60]
Cow	CDMS vs. nLC-MS/MS	Detected 57,350 particles in 8 distinct subpopulations (2D Gaussian model). nLC-MS/MS data corroborated exosome enrichment in CDMS samples and identified 162 proteins and 43 exosome-specific proteins.	[131]
Cow	nLC-MS/MS	Identified 1330 proteins (118 unique to infection) in bovine leukaemia virus (BLV)-infected cattle. Presented 3 proteomic datasets of milk-derived EVs from healthy and BLV-infected cattle.	[139,140]
Cow	nLC-MS/MS	Identified 1899 proteins (20 and 41 specific to 35 K and 100 K pellets, respectively).	[132]
Cow	nLC-MS/MS	A novel subset of EVs with unique proteins and other cargoes.	[133]
Camel	nLC-MS/MS	Identified 1010 functional groups of proteins. Total of 890 proteins in all 3 species, with 5 specific to *C. dromedaries*, 31 to *C. bacterianus*, and 12 to hybrid camels.	[134]
Cow + donkey + goat	UHPLC-HRMS	Metabolomic analysis of 5 different pools of fractions obtained from differential centrifugation from 3 different species.	[141]
		**Exosome**	
Human	iTRAQ-labelled, nLC-MS/MS	Total of 70 peptides from 28 proteins in preterm milk exosomes differentially expressed compared to full-term milk exosomes, with 47 upregulated and 23 downregulated.	[135]
Human + Cow	iTRAQ-labelled, nLC-MS/MS	Total of 920 proteins identified with 575 proteins differentially expressed between colostrum and mature milk in both species.	[142]
Cow	nLC-MS/MS	Total of 9430 proteins identified, with 1264, 1404, 963, and 1306 unique proteins (24, 48, and 72 h colostrum and mature milk, respectively).	[136]
Cow	μLC-MS/MS, 2D LC-MS	Insufficient exosomes from saliva and urine for analyses. Validation of TSG101 protein milk and plasma exosomes. Total of 86 proteins unique to milk exosomes and 37 proteins unique to plasma exosomes identified.	[137]
Cow	iTRAQ-labelled, nLC-MS/MS	Total of 2971 proteins identified, of which 1490, 302, and 334 were unique to exosomes, whey, and MFGMs, respectively. Total of 90 exosome proteins were differentially regulated by mastitis.	[143]
Cow	iTRAQ labelled, nLC-MS/MS	Total of 2107 proteins identified. Major MFGM proteins were abundant in exosomes but only represented 0.4% to 1.2% of the total exosomal proteome compared to 15% to 28% of that of the MFGM proteome.	[128]
Pig	Nlc-MS/MS	Total of 2313 peptides from 639 proteins, with 68 novel proteins identified.	[129]
Horse	MALDI-ToF	Identification of exosome-associated proteins, CD81 and CD63, in horse milk.	[144]

The terminology used is based on the reference cited, and this division reflects older thinking and is a “pool” of EVs that are responsible for the effects (μLC-MS/MS, micro-flow liquid chromatography-tandem mass spectrometry; CDMS, charge detection mass spectrometry; iTRAQ, isobaric tags for relative and absolute quantification; MALDI-ToF, matrix-assisted laser desorption ionisation-time of flight; MFGM, milk fat globule membrane; nLC-MS/MS, nano-flow liquid chromatography-tandem mass spectrometry).

**Table 6:** Major findings of studies on the general stability and uptake of mammalian milk-derived EVs.

Species	Findings	Ref.
	**Extracellular vesicle**	
Human	Stability and uptake of natural and synthetic EVs loaded with locked nucleic acid anti-sense oligonucleotides in vitro (PHH, NCI-H460 cell line, and hPSC) and in vivo (mice).	[216]
Cow	The impact of industrial processing on milk EVs’ structural integrity and molecular composition.	[219]
Cow	Cellular internalisation of EVs in vitro (hPAEC and NRCM).	[109]
Cow	Development of non-invasive fluorescent labelling of EVs in vitro (Caco-2 cell line), demonstrating internalisation and co-localisation of labelled EVs.	[220]
Cow	Time-dependent uptake of colostral miRNA, EV proteins, and isomiRs after feeding in vivo (calves).	[221]
Cow	Demonstrated that microwaving, but not autoclaving, agitation, or freezing, reduced miR-220c abundance.	[207]
	**Exosome**	
Human	Resistance of exosomes isolated from preterm human milk to in vitro digestion and internalisation in vitro (HIEC).	[169]
Human	Exosomal protein markers resist degradation by in vitro digestion, pH 4.5, and the uptake of digested and undigested exosomes, based on immunofluorescence imaging of exosomal protein markers in vitro (HIEC).	[171]
Human	Resistance of miRNA to degradation caused by incubation at 26 °C over 24 h, six freeze–thaw cycles at −20 °C, treatment with RNase A and RNase T1, and incubation at 100 °C for 10 min.	[173]
Human	Demonstrated the uptake of RNA ex vivo (macrophages).	[217]
Human + Cow	Storage at 4 °C substantially reduced the exosome content, especially miRNA, of human milk over time, and the infant formulae tested had no detectable miRNA.	[167]
Cow	Assessed the accumulation and effects of milk exosomes and miRNA cargoes on embryo development in C57BL/6 mice.	[222]
Cow	Resistance of lncRNA to degradation by in vitro digestion.	[177]
Cow	Resistance of paclitaxel (chemotherapeutic), encapsulated in these exosomes, to degradation and loss of efficacy from long-term storage at −80 °C for 4 weeks.	[213]
Cow	Resistance of 5 miRNAs to degradation by an in vitro digestion method and in vitro internalisation of exosomes.	[159]
Cow	Uptake of exosomes and exosome-encapsulated siRNA (both digested and undigested) in vitro (Caco-2 cell line).	[208]
Cow	Fermentation of milk exosomes with probiotic *Streptococcus thermophiles*, Lactobacilli, and Bifidobacteria reduces miR-29b and miR-21 abundance and total protein concentration.	[201]
Cow	Challenged the findings from a previous study [160] regarding the dietary transfer of cow milk-derived miRNA in humans.	[214]
Cow	Demonstrated that miR-223 and miR-125b persist in high abundance after simulated in vitro digestion (TNO TIM-1 model). Authors found that exosomes may not be the only carrier of these miRNAs in milk.	[211]
Cow	Uptake and bioavailability of fluorescent-labelled exosomes and their miRNA cargoes via endocytosis in vivo (C57BL/6 mice) and in vitro (HUVEC).	[210]
Cow	Resistance of native miRNA and anticancer compounds encapsulated in these exosomes to degradation from long-term storage at −80 °C for 6 months.	[212]
Cow	Uptake of miRNA in differentiated and undifferentiated THP-1 cells.	[178]
Cow	Uptake and transport of miRNA by endocytosis in vitro (Caco-2 and IEC-6 cell lines).	[209]
Cow	Uptake of miR-29b and miR-200c in a randomised crossover feeding study, in C57BL/6J mice (± miRNA depletion), and human peripheral blood mononuclear cells (PBMCs).	[160]
Cow + Pig + Mice	Cross-species biodistribution profile of miRNAs in mice and pig model.	[223]
Goat + cancer cell lines	A novel approach of covalently labelled exosomes with commercial fluorophores in vitro (U87 and B16F10 cell lines) and in vivo (C57BL/6 mice).	[224]
Goat	Uptake, bioavailability, and tissue distribution of radiolabelled (reduced technetium, ^99m^Tc (IV)) exosomes using non-invasive single-photon emission computed tomography imaging in Balb/C mice.	[218]
	**Microvesicles/Nanovesicles/Other**	
Human	Presence of immune-related miRNA in human milk, two of which were present in exosomes. miR-21 and miR-181a were resistant to degradation by RNase, pH 1, and freeze–thaw, indicating an extracellular protective mechanism.	[202]
Cow	Pasteurisation and homogenisation, but not 4 °C storage, substantially reduce the abundance of miR-200c and miR29b in four types of milk tested. Somatic cells in the milk accounted for <1% of the abundance of these miRNAs in milk, consistent with these miRNAs packaged in extracellular structures such as EVs.	[215]
Cow	Presence of mRNA and miRNA which were resistant to degradation by RNase, pH 2, incubation at 37 °C, but not Triton X-100, indicating an extracellular protective mechanism.	[206]
Cow	Presence of mRNA and miRNA in both samples. These RNAs were resistant to degradation by pH 2, indicating an extracellular protective mechanism.	[204]
Buffalo	Demonstrated that 4 °C storage and multiple freeze–thaws reduced the abundance of miR-21 and miR-500.	[205]

The terminology used is based on the reference cited, and this division reflects older thinking and is a “pool” of EVs that are responsible for the effects (HIEC, human intestinal epithelial crypt-like cell; hPAEC, human pulmonary artery endothelial cell; hPSC, human pluripotent stem cell; HUVEC, human umbilical vein endothelial cell; NRCM, neonatal rat cardiomyocyte; PBMC, peripheral blood mononuclear cell; PHH, primary human hepatocyte).

## Data Availability

Not applicable.

## Supplementary Materials

The following are available online at https://www.mdpi.com/article/10.3390/nu13082505/s1, Table S1: Total world milk production quantity (FAOSTAT: 2009 and 2019). Table S2: Raw data extracted from different food composition databases.
